# Cellular Communication Networks Mediated by Microglia in Ischemic Stroke

**DOI:** 10.1002/cns.70687

**Published:** 2025-12-09

**Authors:** Feiyu Ma, Yi Bai, Na Li, Xiangyu Cai, Ruicong Xu, Xiang Cao

**Affiliations:** ^1^ Department of Neurology, Nanjing Drum Tower Hospital, Joint Institute of Nanjing Drum Tower Hospital for Life and Health, College of Life Science Nanjing Normal University Nanjing China; ^2^ Department of Blood Transfusion Medicine, School of Medicine, Jinling Hospital Nanjing University Nanjing China; ^3^ Department of Neurology, Nanjing Drum Tower Hospital, Affiliated Hospital of Medical School Nanjing University Nanjing China; ^4^ Nanjing Neurology Clinical Medical Center Nanjing China

**Keywords:** extracellular vesicles, intercellular communication, ischemic stroke, microglia, neuroinflammation, oxidative stress

## Abstract

**Introduction:**

Microglia, the resident immune cells of the central nervous system, rapidly activate after ischemic stroke and actively communicate with neurons, astrocytes, endothelial cells, and infiltrating peripheral immune cells. As ischemic stroke remains a major cause of death and long‐term disability worldwide, growing evidence highlights that microglia‐driven communication—through direct cell–cell contact, soluble factors, and extracellular vesicles—plays a central role in regulating neuroinflammation and shaping disease progression. A clearer understanding of these communication networks may help identify new therapeutic strategies targeting glial function.

**Methods:**

This review summarizes recent advances in understanding microglial states after ischemic stroke and their communication with neural and peripheral immune cells. Literature was collected from PubMed and Web of Science, with attention to mechanisms involving direct cell–cell interaction, cytokine and chemokine signaling, extracellular vesicle communication, and newly described tunneling structures. Key regulatory processes at different pathological stages are compared.

**Results:**

Experimental and clinical evidence shows that microglia display dynamic and heterogeneous activation patterns after ischemic stroke. Through diverse communication pathways, they influence neuronal survival, synaptic remodeling, inflammatory responses, and blood–brain barrier integrity. Soluble mediators—including cytokines, chemokines, and damage‐associated molecular patterns—shape both local and systemic immune reactions, while extracellular vesicles regulate neuroinflammation and tissue repair by transferring bioactive molecules. Recently reported microglial tunneling structures further increase the complexity of intercellular communication. Together, these pathways determine the progression of ischemic injury and recovery.

**Conclusions:**

Microglia act as central coordinators of communication among neurons, glial cells, and immune cells during ischemic stroke, thereby influencing disease severity and functional outcome. Clarifying microglia‐mediated communication mechanisms may help guide the development of targeted immunomodulatory treatments. Continued research will be important for advancing these findings toward clinical translation.

## Graphical abstract

After ischemic stroke, microglia serve as central regulators within the neuroimmune network, engaging in dynamic crosstalk with neurons, astrocytes, vascular endothelial cells, and peripheral immune cells. By releasing cytokines, chemokines, extracellular vesicles, and transmitting mechanical signals, activated microglia coordinate inflammatory responses and tissue repair, underscoring their pivotal role in post‐stroke brain remodeling.

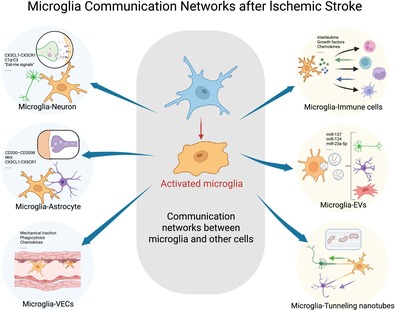

## 1 Introduction

Ischemic stroke, a prevalent cerebrovascular disorder, is the second leading cause of death worldwide and a major contributor to long‐term disability [1]. Its pathophysiology is complex, involving neuroinflammation, oxidative stress, and excitotoxicity [2, 3]. When cerebral arteries become obstructed by thrombosis or embolism, the affected brain regions suffer from reduced oxygen and nutrient supply, disrupting cellular metabolism. Within a short time, neurons and glial cells sustain damage, ionic imbalances develop, and excessive release of excitatory amino acids triggers cascades that ultimately lead to cell death and neurological dysfunction [4]. Although treatments such as intravenous thrombolysis and endovascular thrombectomy exist, their application is limited by narrow therapeutic windows and strict eligibility criteria, leaving many patients without effective intervention [2, 5, 6].

Recent advances in intercellular communication have highlighted its importance in the pathogenesis and potential treatment of ischemic stroke. Microglia, the resident immune cells of the central nervous system, are highly sensitive to cerebral ischemia and respond rapidly following stroke onset. Microglia‐mediated inflammatory responses are now recognized as a key pathological hallmark of ischemic stroke [7]. Upon activation, microglia polarize into distinct phenotypes with divergent functions: the M1 (pro‐inflammatory) phenotype releases cytokines such as interleukin‐1β (IL‐1β), interleukin‐6 (IL‐6), and tumor necrosis factor‐α (TNF‐α), amplifying neuroinflammation and tissue injury; in contrast, the M2 (anti‐inflammatory) phenotype promotes tissue repair and neuroprotection through the secretion of anti‐inflammatory mediators [8, 9]. Although the binary classification is a simplification of microglial states, it provides a convenient framework for research. In fact, recent advances in microglial biology have largely been built upon this M1/M2 paradigm [10, 11, 12]. This phenotypic plasticity allows microglia to dynamically regulate the post‐stroke microenvironment.

In addition to cytokines, activated microglia also secrete chemokines such as C‐C motif chemokine ligand 2 (CCL2), which recruit peripheral immune cells to the injury site, as well as neurotransmitters like glutamate that influence neuronal signaling [13, 14]. These molecules serve as messengers, mediating dynamic communication between microglia and surrounding neurons, astrocytes, and other cell types. Microglia exert their regulatory functions through multiple forms of cell‐to‐cell communication. These include direct contact with neighboring cells, the release of extracellular vesicles carrying bioactive molecules such as proteins, nucleic acids, and lipids, and the formation of tunneling nanotubes, which enable the transfer of organelles and signaling molecules [15, 16, 17].

This review focuses on the role of microglia‐mediated intercellular communication in ischemic stroke, aiming to advance our understanding of the cellular and molecular mechanisms underlying stroke pathology. These insights may help guide the development of targeted therapeutic strategies to improve patient outcomes.

## 2 Direct Interactions Between Microglia and Other Cells

### 2.1 Microglia and Neurons

After ischemic stroke, microglia are rapidly activated, migrate to the injury site, and gradually form elongated protrusions that allow direct contact with neuronal structures such as soma and dendrites. Numerous studies have shown that microglial phagocytosis of stressed neurons leads to neuronal loss, particularly in the ischemic penumbra. Inhibition of this phagocytic process has been shown to improve stroke prognosis [18, 19]. Interestingly, microglia show a preference for re‐establishing contact with previously visited neuronal sites [20].

The complement system becomes overactivated after stroke, with Complement Component 1q (C1q) and complement component 3 (C3) rapidly accumulating around neurons and tagging damaged synapses within 24 h. Subsequently, microglial complement receptor 3 (C3R) recognizes the C3‐tagged synapses and mediates their phagocytosis, leading to synaptic loss and neuronal death [21]. Suppressing the expression of C1q and C3 can attenuate synapse clearance by microglia, thereby reducing brain damage and improving neurological function [22].

**FIGURE 1:**
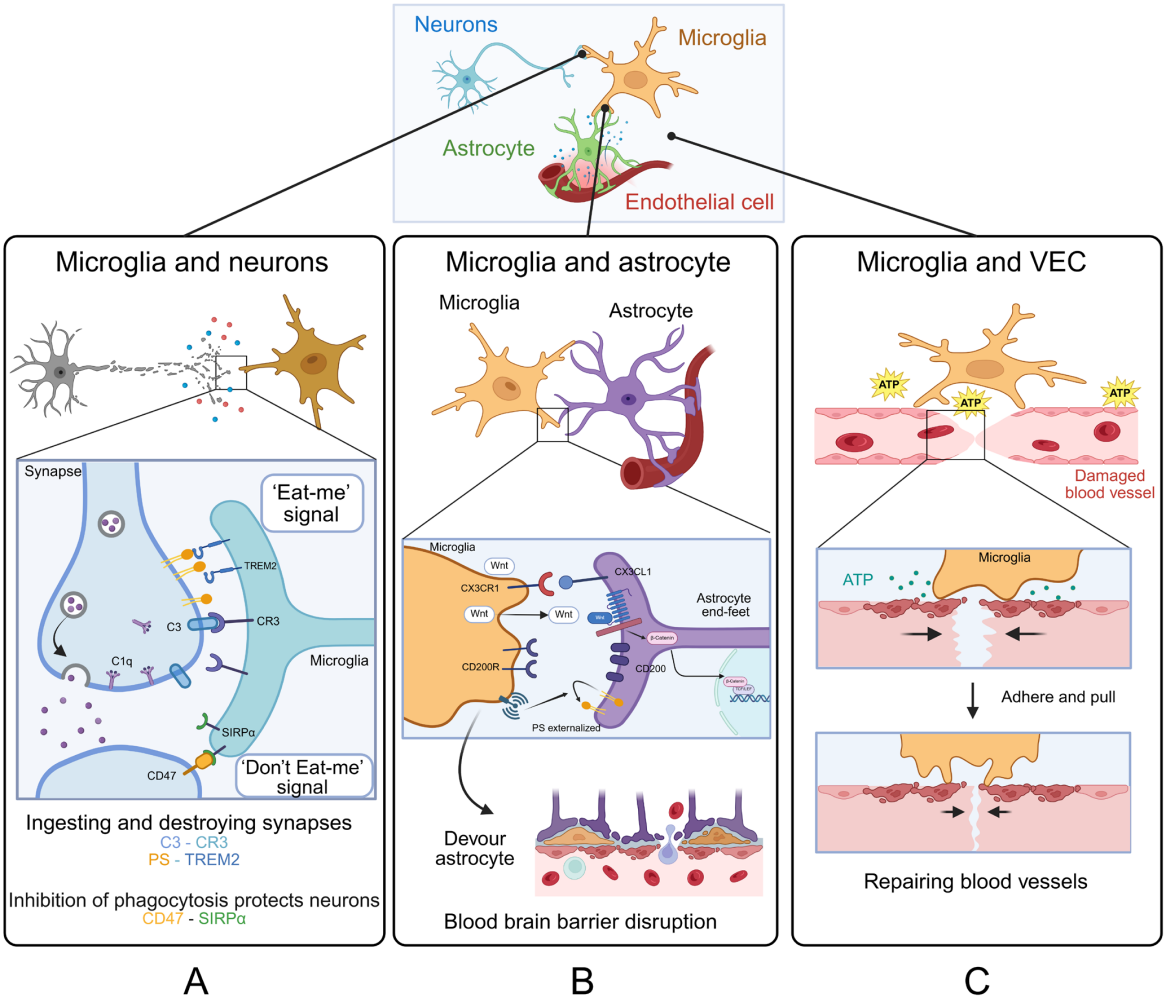
The intercellular communication between microglia and other cells in ischemic stroke.

In addition, microglia actively participate in the programmed neuronal death. Under pathological conditions, neurons expose “eat‐me” signals such as phosphatidylserine (PS) and calreticulin on their surface. PS, normally located on the inner leaflet of the cell membrane, flips to the outer surface upon injury and is recognized by specific microglial receptors [23]. Triggering receptor expressed on myeloid cells 2, a microglial immunoreceptor, binds to PS and regulates synaptic remodeling in regions such as the hippocampus and dorsolateral geniculate nucleus [24, 25]. In contrast, “don't eat me” signals like cluster of differentiation 47 (CD47) inhibit phagocytosis by interacting with signal regulatory protein α on microglia [26]. When synapses are damaged, the expression of CD47 is downregulated, facilitating microglial‐mediated synapse elimination (Figure 1A) [27].

Fluorescent labeling studies have shown that under normal physiological conditions, microglia surveil neurons for approximately 5 min. After ischemic stroke, they maintain contact with neurons for up to 1 h, with increased contact area around synapses. This is accompanied by the loss of presynaptic structures, suggesting that microglia may regulate and protect the synapse by phagocytosis of neuronal components [28, 29]. Csaba et al. found that microglial P2Y12 receptor (P2Y12R) and neuronal cytosol can form special somatic purinergic junctions in the ATP‐rich local space of the neuronal cytosol. These specialized junctions enable continuous contact and monitoring of neuronal status. Post‐stroke, microglial coverage over neurons increases, and microglia mitigate neuronal damage by regulating intracellular calcium ion concentrations and enhancing mitochondrial activity [30].

Ischemic stroke frequently leads to white matter damage, resulting in cognitive and sensorimotor deficits [31]. C‐X3‐C motif chemokine ligand 1 (CX3CL1), also known as fractalkine, is a membrane‐bound chemokine specifically expressed in neurons, while its receptor CX3CR1 is highly enriched in microglia. This axis plays a central role in neuron–microglia communication. Neuron‐derived CX3CL1 binds to microglial CX3CR1 and helps maintain microglia in a homeostatic state, thereby reducing inflammatory damage [32]. Postsynaptic density protein 93 (PSD‐93), a scaffold protein localized in the postsynaptic compartment, has been shown to directly bind to CX3CL1, thereby promoting the cleavage and release of its soluble form. The increased soluble CX3CL1 then binds CX3CR1 on microglia and enhances pro‐inflammatory activation [33]. To disrupt this pathological interaction, a cell‐permeable peptide named Tat‐CX3CL1 was designed based on the CX3CL1 sequence responsible for PSD‐93 binding (amino acids 357–395). Tat‐CX3CL1 competitively blocks the PSD‐93–CX3CL1 interaction, attenuates microglial release of IL‐1β and TNF‐α, shifts microglia from a pro‐inflammatory M1 state to an anti‐inflammatory M2 phenotype, reduces infarct volume, and improves neurological outcomes in ischemic stroke models [34]. These findings suggest that targeting neuron‐derived CX3CL1 signaling via PSD‐93 may represent a novel strategy for regulating microglia‐mediated neuroinflammation after stroke.

### 2.2 Microglia and Astrocytes

Astrocytes account for approximately 19%–40% of neuroglial cells in the central nervous system (CNS) and play a crucial role in maintaining brain homeostasis [35]. Under normal conditions, they contribute to neuromodulation, the integrity of the blood–brain barrier (BBB), and support synaptic function. However, following ischemic injury, astrocytes become reactive and transform into a pro‐inflammatory phenotype, commonly referred to as “reactive astrocytes”. These activated astrocytes are predominantly found in the ischemic penumbra and actively participate in tissue repair, secretion of neurotrophic factors, and inflammatory responses [36].

Microglia and astrocytes can directly interact through physical contact and molecular signaling. For instance, following cerebral ischemia, PS externalization is observed not only in necrotic neurons but also on the surface of astrocytes. Microglia are able to recognize and engulf these PS‐externalized astrocytes, contributing to BBB disruption. Interestingly, targeting PS on astrocytes using membrane‐conjugated protein Annexin A5 inhibits microglial phagocytosis, thereby attenuating BBB breakdown and offering a potential therapeutic approach to protect the vascular integrity after ischemic stroke [37].

Cluster of Differentiation 200 (CD200), a transmembrane glycoprotein, is expressed on the surface of neurons, endothelial cells, and astrocytes, while its receptor, CD200R, is primarily expressed on microglia. CD200R signaling plays a crucial role in regulating microglial activation. In CD200‐deficient mice, microglial activation is exacerbated, leading to an enhanced neuroinflammatory response. Studies have shown that astrocytes can activate microglia through the CD200‐CD200R axis, further enhancing cellular communication between the two glial populations [38]. Recent research by Bravo et al. demonstrates that neuron–microglia crosstalk can be mediated through astrocyte‐dependent mechanisms, wherein upregulation of the CD200‐CD200R axis specifically modulates microglial reactivity. This finding reveals a novel tripartite communication mechanism among neurons, astrocytes and microglia [39, 40].

In addition to the CD200‐CD200R interaction, the CX3CL1‐CX3CR1 axis also plays an important role in the communication between microglia and astrocytes. Recent studies have shown that microglia release Wnt signaling molecules through the CX3CL1‐CX3CR1 axis, which activates the Wnt receptor in astrocytes. This signaling results in a reduction in synaptic contacts within astrocytes, creating a favorable environment for synaptic phagocytosis by microglia. However, in CX3CL1‐deficient mice, this reduction in synaptic contacts was not observed, suggesting that the CX3CL1‐CX3CR1 signaling pathway is essential for this interaction between microglia and astrocytes (Figure 1B) [41].

Astrocytes exhibit a dual role in cerebral ischemia. During the early stages, reactive astrocytes can exacerbate neuronal damage by promoting the release of inflammatory mediators, primarily through the phosphorylation of connexin 43, which leads to the degradation of gap junctions and the opening of hemichannels [42]. However, in the later stages of ischemic injury, sustained activation of microglia induces the reactive proliferation of astrocytes, leading to the formation of a glial scar in the peri‐infarct region. This glial scar serves to limit the spread of inflammation and attenuate neuroinflammatory injury [43].

### 2.3 Microglia and Vascular Endothelial Cells

Vascular endothelial cells (VECs) are critical components of the BBB, maintaining its structural integrity and regulating the exchange of molecules between the blood and the brain parenchyma [44]. Microglia significantly modulate endothelial cell function and BBB integrity after ischemic stroke. In stroke models, the presence of CD31 granules and other endothelial markers correlates with CD68 phagosomes in perivascular microglia, suggesting that microglia phagocytose endothelial cells post‐stroke, contributing to BBB disruption. Additionally, microglia‐derived TNF‐α exacerbated oxygen–glucose deprivation (OGD)‐induced endothelial necrotic cell death in primary co‐cultures of microglia and endothelial cells using the Transwell system [45, 46].

Microglia also participate in post‐injury revascularization through physical interactions. In zebrafish models of cerebrovascular injury, microglia are rapidly activated and migrate to the injury site. Damaged vessels secrete ATP, which recruits microglia, causing them to extend pseudopods and adhere to the severed ends of the vessels. Once microglia and endothelial cells form new adhesive connections, microglia mechanically pull the severed ends closer, ultimately facilitating vascular reconnection (Figure 1C) [47].

Endothelial cells and their progenitors actively regulate microglial activity during ischemic stroke. Endothelial progenitor cells (EPCs), mobilized from the bone marrow to ischemic regions, differentiate into new VECs and promote angiogenesis. Importantly, EPCs have been shown to modulate microglial activity. For instance, EPC transplantation reduces astrocyte‐derived C3/C3aR pathway activation, promotes microglial polarization toward an anti‐inflammatory phenotype, and enhances C3R‐mediated phagocytosis, ultimately attenuating synaptic loss and improving neurological recovery after ischemic stroke [48]. VECs also influence microglial behavior through the release of signaling molecules. For example, VECs promote leukocyte recruitment through the production of chemokines, which facilitate direct interactions between endothelial cells and leukocytes, triggering an inflammatory response that further activates microglia [49].

Despite these advances, direct interactions between microglia and VECs remain underexplored. Identifying key molecular targets in their interaction could enable the regulation of microglial activity and polarization, potentially reducing inflammatory damage and promoting neuroprotection after ischemic stroke.

### 2.4 Microglia and Oligodendrocytes

Oligodendrocyte progenitor cells (OPCs) are critical cells responsible for myelin production in the central nervous system (CNS). Following the onset of ischemic stroke, OPCs become one of the most vulnerable cell populations due to their high metabolic demands and sensitivity to excitotoxicity, leading to extensive demyelination and white matter damage. To initiate repair, endogenous OPCs are activated and migrate toward the lesion site [50]. However, their differentiation and remyelination processes are often impaired by the inhibitory microenvironment. Microglia exert a dual role in regulating OPC‐mediated repair processes [51].

A population of amoeboid microglia present in the corpus callosum during early development, termed the “microglial fountain,” can directly phagocytose a subset of OPC precursors prior to myelination. CX3CL1‐deficient microglia exhibit reduced phagocytosis of OPCs and decreased myelin thickness, which is crucial for OPCs to regulate myelination [52]. Additionally, during disease progression, resident microglia in the brain also function to clear dying OPCs, and this process is mediated by the Fractalkine‐CX3CR1 axis [53].

A study employing in vivo imaging techniques demonstrated that neuropilin‐1 (Nrp1) expressed on microglia establishes close contacts with the processes of oligodendrocyte progenitor cells (OPCs). This interaction promotes the proliferation of platelet‐derived growth factor AA (PDGF AA), which in turn mediates the proliferation of OPCs in the white matter. Upon binding to its ligand, Nrp1 activates downstream signaling pathways (e.g., the PI3K‐Akt pathway), thereby enhancing the proliferative capacity of OPCs. This mechanism carries significant implications for neural repair and remyelination following cerebral ischemia [54].

After ischemic stroke, microglia interact with neurons, astrocytes, and vascular endothelial cells in the brain. They influence the blood–brain barrier and angiogenesis through cell–cell contacts or direct mechanical action.

## 3 Indirect Communication via Soluble Signaling Molecules

### 3.1 Interleukins

Interleukins (ILs) play a crucial role in regulating inflammation during ischemic stroke. They are involved in both initiating and exacerbating the inflammatory response, as well as in neuroprotection and tissue repair.

IL‐6 is a pleiotropic cytokine that plays an important role in angiogenesis, neuroprotection, and functional recovery in a mouse model of stroke. Elevated IL‐6 levels have been linearly correlated with an increased long‐term risk of ischemic stroke [55, 56, 57]. While traditionally considered pro‐inflammatory, recent studies suggest that IL‐6, produced by microglia, also aids in neural regeneration and hippocampal repair after brain injury [58]. Notably, recent studies have demonstrated that IL‐6 is essential for the generation of repair‐associated microglia in the ischemic penumbra, which promote vascular repair and neuronal recovery [59]. These findings highlight IL‐6 as a potential therapeutic target for ischemic stroke.

Following cerebral ischemia, damaged microglia release IL‐33, a potent chemokine that enhances neuronal survival and preserves white matter integrity through the IL‐33/ST2 signaling pathway. Studies have shown that IL‐33 enhances the release of IL‐10 from microglia, which is essential for the protection of brain neurons as well as for the repair of the blood–brain barrier [60]. In addition, IL‐33‐treated microglia mitigate oligodendrocyte and oligodendrocyte precursor cell (OPC) death under OGD, promoting OPC differentiation, myelination, and functional white matter recovery after stroke [61].

Another emerging cytokine, IL‐38 (a member of the IL‐1 family), inhibits NOD‐like receptor thermal protein domain associated protein 3 (NLRP3) inflammasome activation in microglia, reducing inflammation and protecting VECs from ischemic damage [62].

As both the primary source and key responder to interleukin signaling, microglia not only secrete them to modulate neuronal and vascular functions, but also undergo phenotypic shifts when exposed to these mediators. This reciprocal interaction creates a cytokine‐microglia amplification loop, where cytokine‐stimulated microglia further release secondary inflammatory/regenerative factors that influence oligodendrocytes, endothelial cells, and neurons. Targeting this dynamic interplay may provide novel therapeutic opportunities for stage‐specific intervention in ischemic stroke.

### 3.2 Growth Factors

Growth factors play multifaceted roles in neuroregeneration, angiogenesis, and inflammation modulation following ischemic stroke. Vascular endothelial growth factor promotes post‐ischemic vascular and neuronal regeneration by regulating autophagy and cellular metabolism, while facilitating the migration of newborn neurons to ischemic areas [63, 64]. Fibroblast growth factor primarily enhances neovascularization in brain tissue to improve local blood supply, though its clinical translation remains exploratory.

Mesencephalic astrocyte‐derived neurotrophic factor (MANF), an endoplasmic reticulum stress‐related protein initially identified in midbrain astrocytes, shows significantly increased secretion from neurons after cerebral ischemia and relocates to activated microglia. Exogenous MANF can modulate microglial function by reducing pro‐inflammatory factors and elevating IL‐10 expression, thereby diminishing infarct volume [65]. Brain‐derived neurotrophic factor (BDNF) promotes axonal regeneration via the TrkB signaling pathway. Meanwhile, microglia‐derived BDNF suppresses their own overactivation through the BDNF‐hNSC‐Exo pathway, mitigating neuroinflammation [66].

The CSF‐1/CSF‐1R axis is pivotal in neuron–microglia communication. Neurons in peri‐infarct regions markedly upregulate CSF‐1R post‐ischemia [67, 68]. While the CSF‐1R inhibitor PLX3397 effectively depletes microglia, it transiently exacerbates astrocyte‐mediated inflammation [69, 70, 71]. Notably, newly repopulated microglia after drug withdrawal polarize toward an anti‐inflammatory phenotype, enhancing blood–brain barrier integrity and elevating BDNF levels, ultimately improving outcomes [72].

### 3.3 Chemokines

Crosstalk between the nervous and immune systems has received increasing attention in recent years due to its emerging role in neurological disorders. Following stroke, various peripheral immune cells are successively recruited by chemokines secreted by microglia, which arrive in the brain within one day, peak in number within three days, and initiate a robust neuroinflammatory response. Lymphocytes, for example, inhibit M1‐type microglial activation by suppressing NF‐κB signaling, while their own migration is also regulated by microglia‐derived chemokines. Transwell migration assays have shown that regulatory T cells (Tregs) can migrate toward the medium of OGD‐activated microglia, with microglia‐derived CCL22 and its receptor C‐C chemokine receptor type 4 (CCR4) being key mediators. CCR4 overexpression significantly increases Treg recruitment and reduces infarct size [73]. Similarly, CD8^+^ T cells are recruited via microglia‐mediated CCL2/CCL8 and CCR2/CCR5 chemotaxis, but unlike Tregs, they exacerbate brain injury by releasing perforin and granzymes [13].

Neutrophils are the earliest immune cells to infiltrate the ischemic brain, where they contribute to neuronal damage by releasing neutrophil extracellular traps. Our group has shown that lncRNA U90926 expression is elevated in microglia after stroke and promotes neutrophil infiltration by competitively binding to malate dehydrogenase 2 (MDH2), which stabilizes CXCL2 mRNA. Inhibiting U90926 improves stroke prognosis [74].

Chemokine signaling also mediates white matter repair and demyelination. Astrocyte‐derived CXCL5 is upregulated in ischemic brains and impairs remyelination by binding to CXCR2 receptors on microglia and suppressing myelin debris clearance [75, 76]. CKLF1, a CC‐type chemokine, promotes M1 polarization of microglia through NF‐κB‐dependent transcription and activation of the p38 and JNK pathways, contributing to ischemic injury [77].

In addition to classical chemokines, recent studies have identified alternative signaling pathways through which peripheral immune cells modulate microglial activity. Notably, Liu et al. demonstrated that properdin, a complement system protein released by infiltrating neutrophils and myeloid cells, markedly enhances microglia‐mediated neuroinflammation. Mechanistically, properdin binds to the macrophage‐inducible C‐type lectin (Mincle) receptor on microglia, triggering robust pro‐inflammatory signaling and exacerbating neuronal damage [78]. Specific T cell subsets have also been shown to modulate microglial activation and polarization after stroke. Our previous studies demonstrated that double‐negative T cells markedly promote neuroinflammation and aggravate ischemic injury by enhancing pro‐inflammatory microglial responses [79]. In another study, we found that genetic or pharmacological inactivation of FasL signaling in CD4^+^ T cells alleviates microglia‐mediated neurotoxicity by promoting a shift toward the M2 anti‐inflammatory phenotype [80]. These findings underscore the critical role of peripheral cells in shaping microglial function and highlight microglia as key mediators of peripheral–central immune crosstalk in ischemic stroke.

## 4 Hormonal Regulation of Microglial Activity

After ischemic stroke, microglia sense and respond to hormonal signals through surface receptors, thereby mediating intercellular communication. These hormonal signals primarily originate from two sources: endogenous fluctuations induced by ischemic injury and exogenous interventions applied therapeutically, and their regulatory effects on microglia are context dependent. For example, estrogen promotes microglial polarization toward the anti‐inflammatory M2 phenotype [81]. This phenotypic shift enhances microglial phagocytosis and reduces the release of pro‐inflammatory cytokines, indirectly supporting neuronal survival and facilitating astrocytic repair, highlighting hormone‐driven microglia‐to‐cell communication during brain recovery.

Melatonin, an endogenous hormone secreted by the pineal gland, exerts neuroprotective effects by modulating microglial activity [82]. It crosses the blood–brain barrier and inhibits M1 polarization via the SIRT1/Nrf2 and MAPK/ERK pathways, reducing neuroinflammation and neuronal apoptosis [83, 84]. Catecholamines such as norepinephrine and epinephrine, whose levels are mostly elevated endogenously by ischemic stroke (with exogenous interventions rarely used clinically due to side effects), signal through adrenergic receptors on microglia, showing both anti‐inflammatory and pro‐inflammatory effects depending on the context [85, 86]. β‐adrenergic receptor activation can suppress NF‐κB signaling and IL‐1β production, while excessive stimulation may enhance cytokine release [87, 88, 89, 90]. Glucocorticoids, which exhibit both ischemia‐induced endogenous changes and therapeutic exogenous interventions, through glucocorticoid receptors expressed on microglia, broadly suppress neuroinflammatory responses. Loss of microglial GRs leads to increased IL‐1β and TNF‐α levels in ischemic brain tissue [91, 92]. Recent single‐cell RNA sequencing studies by our team identified a glucocorticoid‐induced microglial subpopulation—ischemia‐associated protective microglia—which exhibits reduced pro‐inflammatory gene expression compared to core‐associated microglia, highlighting a distinct transcriptional profile associated with neuroprotection [10].

Overall, hormones modulate microglial phenotype and function in a way that shapes their interaction with neurons, glia, and vascular cells. This endocrine–immune interface represents a crucial layer of microglia‐mediated cell‐to‐cell communication in ischemic stroke, and targeting this axis may offer novel strategies for neuroprotection and individualized therapy.

## 5 Neurotransmitter–Microglia Interactions

Neurotransmitters serve as key intermediaries in microglia‐mediated interactions with surrounding neurons and glial cells. Microglia express a wide range of neurotransmitter receptors, enabling them to sense synaptic activity and modulate their behavior accordingly. γ‐aminobutyric acid (GABA), through GABAB receptors on microglia, inhibits NF‐κB and MAPK signaling, reducing pro‐inflammatory cytokine production and promoting M2‐like polarization [93, 94]. GABA also facilitates selective pruning of inhibitory synapses by microglia, promoting synaptic remodeling and reducing network hyperexcitability. In addition to its trophic roles described earlier, BDNF, released from microglia, acts in a feedback loop to regulate synaptic integrity through ERK1/2 and GSK3β signaling, thereby modulating the balance between excitatory and inhibitory transmission [95, 96].

Purines are another class of neurotransmitter, and binding to purinergic receptors is another major axis of microglial responsiveness to ischemic injury [97]. While P2Y12 receptors contribute to neuronal surveillance in the healthy brain, they also mediate microglial chemotaxis toward ATP released from injured cells, enabling a rapid response to neuronal stress [98]. P2Y6 receptors are essential for microglial engulfment of apoptotic neurons, and P2X4 receptors regulate phagocytic function through modulation of scavenger proteins like CD36 [99]. Excessive ATP stimulation activates the P2X7 receptor–THIK‐1–Na^+^/K^+^‐ATPase complex, triggering NLRP3 inflammasome assembly and pro‐inflammatory cytokine release [100, 101]. This purinergic cascade links microglial activation to neuron‐derived danger signals and directly influences the inflammatory microenvironment.

Acetylcholine, acting through α7 nicotinic acetylcholine receptors (α7nAChR) on microglia, modulates inflammatory output in response to cholinergic tone. Vagus nerve stimulation enhances Acetylcholine release and upregulates α7nAChR expression, suppressing NF‐κB activity and NLRP3 inflammasome assembly [102, 103]. This pathway attenuates microglia‐derived IL‐1β and Caspase‐1 production, thereby protecting neurons from secondary inflammatory damage [104, 105]. Neurotransmitter–microglia interactions thus serve as dynamic modulators of intercellular signaling in the ischemic brain, and targeted interventions along these axes may offer promising therapeutic strategies.

## 6 Extracellular Vesicle‐Mediated Communication

**FIGURE 2:**
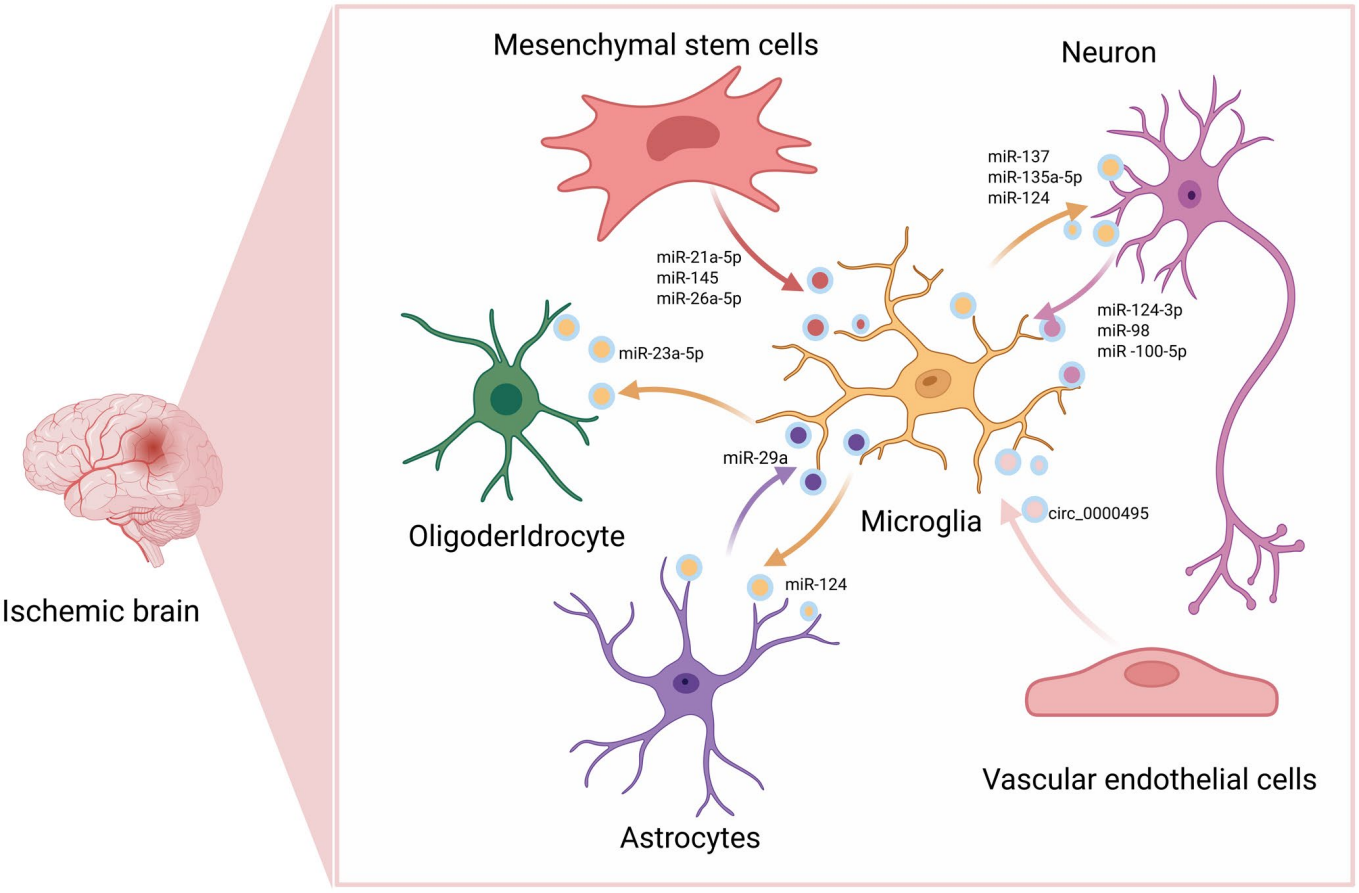
Cellular communication between microglia and extracellular vesicles.

In the CNS, extracellular vesicles (EVs) are nanoscale membrane‐bound particles containing bioactive molecules such as proteins, RNAs, and lipids. EVs can cross the BBB and mediate intercellular communication, playing essential roles in both physiological and pathological processes. Recent studies suggest that modulating the molecular contents of EVs, particularly microRNAs (miRNAs), may improve cellular communication and offer neuroprotective effects after cerebral ischemia, presenting a promising therapeutic target for ischemic stroke [106]. Current research on the transmission of EVs between microglia and other cells is shown in Table 1 and Figure 2.

**TABLE 1:** Effects of EVs between microglia and other cells on ischemic brain injury.

Source	Cargo	Influence	Mechanism of action/signal pathway	Quote
M2‐EVs	miR‐137	Inhibit the apoptosis of nerve cells	Directly targeting Notch1	[107]
M2‐EVs	miR‐135a‐5p	Promote the proliferation of neuron cells	Inhibition of TXNIP expression and activation of NLRP3 inflammatory corpuscles	[108]
M2‐EVs	miR‐124	Inhibit the proliferation and migration of astrocytes	Inhibit the STAT3 signaling pathway	[109]
M2‐EVs	miR‐124	Regulating the proliferation and differentiation of neural stem cells	AAK1/NOTCH signaling pathway	[110]
M2‐EVs	miR‐23a‐5p	Promoting oligodendrocyte production and affecting white matter repair	Directly targeting Olig3	[111]
Microglia	TGF‐β1	Inducing microglia to polarize to M2 type	Activating TGF‐β/Smad2/3 signal pathway	[112]
VECs‐Exos	circ_0000495	Inducing microglia to polarize to M1 type	Activating TLR4/NF‐κB signal pathway	[113]
ADEVs	miR‐29a	Reduce focal death of N9 microglia	Inhibition of NF‐κB/NLRP3 signal pathway	[114]
NDEVs	miR‐124‐3p	Inhibit the activation of M1 microglia and A1 astrocytes.	Inhibit MYH9, regulate the PI3K/Akt/NF‐κB signaling pathway	[115]
NDEVs	miR‐98	Reduce the damage of inflammatory reaction to neurons	Inhibit the phagocytosis of microglia mediated by PAFR and inhibit the expression of iNOS	[116]
NDEVs	miR‐100‐5p	Inducing M1 microglia to produce proinflammatory cytokines	It binds to TLR7 through UUG‐motif and activates NF‐κB pathway.	[117]
MSC‐EVs	miR‐21a‐5p	Inducing microglia to polarize to M2	Phosphorylation of targeted STAT3	[118]
MSC‐EVs	miR‐145	Inducing microglia to polarize to M2	Down‐regulation of FOXO1 inhibits apoptosis, cell cycle arrest and oxidative stress in BV2 cells.	[119]
MSC‐EVs	miR‐26a‐5p	Inhibition of microglia apoptosis	Inhibition of CDK6 expression	[120]

### 6.1 Effects of Microglia‐Derived EVs on Other Cells

Microglia‐derived EVs serve as important vehicles for microglia‐to‐cell communication in the post‐ischemic brain. For example, the Notch signaling pathway, which regulates cell survival and apoptosis, is closely involved in ischemic neuronal damage. Activation of Notch1 after stroke promotes neuronal apoptosis, whereas its inhibition improves neuronal survival [121]. Zhang et al. demonstrated that M2‐type microglial EVs (M2‐EVs)contain miR‐137, which targets Notch1 and reduces neuronal apoptosis in ischemic stroke models [107]. In addition, miR‐135a‐5p, also enriched in M2‐EVs, downregulates TXNIP expression, suppresses NLRP3 inflammasome activation, and promotes neuronal proliferation and survival [108].

Beyond neuronal protection, M2‐EVs influence astrocyte activity and glial scar formation. Li et al. reported that M2‐EVs carrying miR‐124 inhibit astrocyte proliferation and migration by downregulating STAT3 and phosphorylated STAT3, thereby reducing glial scar formation [109]. The same miRNA also regulates neural stem cell proliferation and differentiation through the AAK1/Notch pathway, contributing to functional recovery after stroke [110]. Moreover, M2‐EVs promote white matter repair by targeting oligodendrocytes. Specifically, miR‐23a‐5p within these EVs targets Olig3, a transcription factor critical for oligodendrocyte function, facilitating remyelination and improving neurological outcomes [111].

In addition to delivering miRNAs, microglia‐derived EVs can carry cytokines such as TGF‐β1. EVs obtained from OGD‐treated primary microglia were shown to activate the TGF‐β/Smad2/3 pathway and promote M2 polarization of surrounding microglia, thus enhancing functional recovery in ischemic brains [112]. EVs may also participate in mitochondrial transfer, delivering intact mitochondria or mtDNA‐containing vesicles to recipient cells with impaired mitochondrial function. This emerging mechanism helps restore metabolic activity and cellular viability, but the specific role of microglia in EV‐mediated mitochondrial exchange remains poorly understood [122]. Further investigation into how microglia modulate neuronal recovery through this process may reveal new therapeutic opportunities for stroke.

### 6.2 Effects of EVs Derived From Other Cells on Microglia

In addition to exporting signals, microglia also receive EVs from other CNS‐resident or peripheral cells, which significantly influence their behavior and function. EVs derived from VECs, for instance, can transfer circ_0000495 to microglia, activating the TLR4/NF‐κB signaling pathway and promoting M1 polarization, thereby aggravating vascular damage [113]. Astrocyte‐derived EVs (ADEVs) carrying miR‐29a inhibit the NF‐κB/NLRP3 pathway, reducing OGD‐induced pyroptosis in microglia and limiting infarct volume [114].

Neuron‐derived EVs (NDEVs) also modulate microglial polarization and function. For example, miR‐124‐3p delivered via NDEVs suppresses MYH9 and regulates the PI3K/Akt/NF‐κB pathway, inhibiting M1 microglia and A1 astrocyte activation, thereby protecting neural tissue [115]. miR‐98 from NDEVs blocks PAFR‐mediated phagocytosis and reduces iNOS expression in microglia, mitigating inflammation‐induced neuronal injury. Conversely, some EVs exacerbate damage [116]; miR‐100‐5p activates TLR7 through the U18U19G20 motif, triggering NF‐κB signaling and inducing M1 microglia‐mediated neurotoxicity [117].

Mesenchymal stem cell (MSC)‐derived EVs have attracted attention for their therapeutic potential. These EVs contain miR‐223‐3p, which suppresses cysteinyl leukotriene expression and limits M1 polarization, thereby alleviating brain injury. When applied to OGD‐stressed BV2 cells, MSC‐EVs increase microglial viability and reduce pro‐inflammatory cytokine expression [118, 119]. Moreover, miR‐21a‐5p in MSC‐EVs promotes M2 polarization by targeting STAT3, while miR‐26a‐5p inhibits cyclin‐dependent kinase 6 (CDK6) and reduces microglial apoptosis. These combined effects highlight the potential of MSC‐derived EVs to modulate microglial phenotype and function, offering multi‐faceted neuroprotection after stroke [120].

Microglia mediate intercellular communication with mesenchymal stem cells, oligodendrocytes, astrocytes, neurons, and vascular endothelial cells in the ischemic brain via extracellular vesicle‐derived microRNAs (e.g., miR‐21a‐5p, miR‐137) and circular RNAs (e.g., circ_0000495), thereby regulating cellular crosstalk in cerebral ischemia.

## 7 Tunneling Nanotubes as Structural Communication Bridges

Tunneling nanotubes (TNTs) are specialized membranous structures composed of actin and microtubules that physically connect adjacent cells, functioning as nanoscale conduits for intercellular communication. Their formation involves cytoskeletal rearrangement, including actin polymerization, membrane protrusion, and the extension of microtubules, ultimately establishing direct cell‐to‐cell bridges [123, 124, 125]. Unlike traditional paracrine signaling mechanisms, TNTs provide a direct, rapid, and selective route for transporting cellular cargo—such as proteins, organelles, and genetic material—between connected cells, bypassing extracellular diffusion, TNTs exhibit a certain degree of expression specificity, which is mainly concentrated between microglia and neurons. Currently, relevant studies have been conducted both in vitro and in vivo [126]. This unique mode of communication plays an increasingly recognized role in CNS physiology and pathology, including after ischemic stroke.

Recent findings revealed that microglia can help remove damaged neuronal material via TNTs, thereby assisting in neuronal recovery and network reconstruction. One critical mechanism is the transfer of healthy mitochondria from microglia to neurons under oxidative stress conditions, which supports neuronal antioxidant capacity and promotes survival. Scheiblich et al. demonstrated that in the co‐culture model of microglia and neurons, when microglia are exposed to pathogenic protein aggregates, they form TNTs to deliver functional mitochondria into stressed neurons, restoring mitochondrial membrane potential and rescuing them from dysfunction and cell death [127]. This finding provides direct evidence that microglia can act as metabolic supporters of neurons via TNT‐mediated organelle transfer in the damaged brain.

TNTs also mediate the transport of pathological proteins such as α‐synuclein (α‐syn), a key molecule in neurodegeneration. α‐syn aggregates exacerbate oxidative stress and neuronal toxicity after stroke [128, 129]. Chakraborty et al. reported that TNTs facilitate the bi‐directional transfer of α‐syn and mitochondria between neurons and microglia, suggesting that microglia can receive pathogenic proteins from neurons for degradation while supplying metabolic support in return [130]. This bi‐directional exchange not only modulates inflammation but also shapes the neuroprotective or neurotoxic outcome of microglial responses in the ischemic brain. Furthermore, in post‐mortem brain tissues of patients with Parkinson's disease, TNT‐like structures between neurons and microglia were observed using focused ion beam scanning electron microscopy. Moreover, α‐synuclein (α‐Syn) aggregates and mitochondria were detected within these TNTs, which is consistent with the conclusions from in vitro studies.

Further supporting the immunological role of TNTs, Zaccard et al. revealed that TNTs are frequently observed at the microglia–neuron interface in neuroinflammatory conditions [131]. Their study highlights that TNT formation is upregulated in inflammatory environments, enhancing direct contact‐dependent communication between microglia and neurons. This evidence suggests that similar mechanisms may be activated during ischemic stroke, where neuroinflammation is a major contributor to disease progression.

Together, these studies underscore the pivotal role of TNTs as direct structural bridges for cell‐to‐cell communication between microglia and neurons. Through the exchange of mitochondria, pathogenic proteins, and possibly RNA or cytokines, TNTs provide a mechanism for microglia to dynamically influence neuronal fate. However, the precise molecular mechanisms governing TNT formation, cargo selectivity, and directionality remain incompletely understood. Future research is needed to better define the therapeutic potential of modulating TNT‐mediated microglial communication in ischemic stroke.

## 8 Summary and Outlook

Ischemic stroke poses a severe threat to human health, and its complex pathophysiological processes have long been a key focus of medical research. During this process, intercellular communication mediated by microglia in the central nervous system plays a crucial role. Microglia interact with surrounding cells through direct contact, indirect signaling, EVs, and TNTs, forming a complex communication network that influences the onset, progression, and outcome of ischemic stroke.

Direct contact relies on surface molecule interactions, allowing microglia to establish close physical connections with neurons and other glial cells. After stroke onset, such contact enables rapid signal transmission and initiates a cascade of cellular responses. Indirect communication mainly occurs through the secretion of cytokines, chemokines, and neurotransmitters, which diffuse to nearby cells and regulate their functions. However, dysregulation of this signaling can lead to excessive production of inflammatory factors, triggering a cytokine storm and aggravating neural tissue damage.

EVs, as key mediators of microglia‐to‐cell communication, have garnered increasing attention in ischemic stroke research. These vesicles contain proteins, nucleic acids, and lipids, and can transfer bioactive signals to distant cells, thereby influencing their fate and function. TNTs represent a newly recognized structure that forms direct physical connections between cells, enabling rapid exchange of information and material. In ischemic stroke, TNTs may facilitate efficient communication between microglia and neurons, participating in cellular repair and inflammatory regulation. Although research on their mechanisms remains limited, existing studies suggest that TNTs play a unique and promising role in post‐stroke recovery.

While significant progress has been made in understanding the roles of microglia‐mediated intercellular communication in ischemic stroke, many questions remain. Mechanistically, although the basic functions of different communication modes have been elucidated, their synergistic or antagonistic interactions, as well as their dynamic changes throughout disease progression, are not yet fully understood. Future studies will benefit from the application of multi‐omics technologies and single‐cell analysis to systematically dissect the regulatory networks underlying microglia‐mediated communication at various pathological stages of stroke. In conclusion, microglia‐driven intercellular communication holds substantial research value and therapeutic potential in ischemic stroke. Continued exploration in this field is expected to yield novel strategies for intervention, ultimately improving patient outcomes and quality of life.

## Author Contributions

All authors contributed to the study conception and design. Conceptualization: Xiang Cao. Writing original draft preparation: Feiyu Ma, Yi Bai, and Na Li. Literature search: Ruicong Xu and Xiangyu Cai. Reviewing and editing: Xiang Cao. All authors have read and agreed to the submitted version of the manuscript.

## Funding

This study was kindly funded by the National Natural Science Foundation of China (Grants 82171310 and 82371326) and the Natural Science Foundation of Jiangsu Province (Grant BK20240118).

## Ethics Statement

The authors have nothing to report.

## Consent

The authors have nothing to report.

## Conflicts of Interest

The authors declare no conflicts of interest.

## Data Availability

Data sharing not applicable to this article as no datasets were generated or analysed during the current study.
